# Supplementary material for: Facilitators and barriers to community-based livestock abortion reporting for Rift Valley Fever surveillance in Uganda: A COM-B analysis
Source: PLoS One. 2026 Oct 8;21(10):e0359872. doi: 10.1371/journal.pone.0359872 (PMC13649006; doi:10.1371/journal.pone.0359872)
Supplement: Supplementary file 2 [file pone.0359872.s002.docx]

**S1 Table. A List of participants trained as part of capacity building efforts**

| **Category** | **Details of participants** | **Number** |
| --- | --- | --- |
| District officials | District Health Officer, District Veterinary Officer, Assistant District Health Officer – Environmental Health, Assistant District Officer – Maternal Child Health, District Laboratory Focal Person, Senior Health Educator, Senior Environmental Health Officer | 7 |
| Sub county Technical Officers | Subcounty chief/Town Clerk, Health Inspector/Health Assistant, Veterinary Officer/Animal Husbandry officers, Community Development Officers, Parish Chiefs/Town Agents | 70 |
| Community Health Workers | Village Health Team members, Community Animal Health Workers | 35 |
| Livestock-engaged community members | Livestock owners, abattoir staff, local council leaders, diary staff | 788 |
| **Total** |  | **900** |
