# Supplementary material for: Facilitators and barriers to community-based livestock abortion reporting for Rift Valley Fever surveillance in Uganda: A COM-B analysis
Source: PLoS One. 2026 Oct 8;21(10):e0359872. doi: 10.1371/journal.pone.0359872 (PMC13649006; doi:10.1371/journal.pone.0359872)
Supplement: Supplementary file 5 [file pone.0359872.s005.docx]

**S5 Appendix. Comparative Analysis and Ranking of Facilitators and Barriers: KII vs FGD Perspectives**

a) **Similarities and Differences Between KII and FGD Participants**

| **Theme** | **KII Perspective** | **FGD Perspective** | **Common Ground** |
| --- | --- | --- | --- |
| **Knowledge gaps as fundamental barrier** | "The problem I see from the district downwards, which we call the community-level; capacity is low. So, in other words; we have capacity upwards, but we don't have capacity downwards because the farmers do not know the disease" (KII-ACAD-1) | "What stops us to curb down the speed of this disease is limited knowledge about the disease" (FGD-LO-5, Nakivale) | **Both groups identify poor RVF knowledge as the primary obstacle** (55 references total, highest of any code) |
| **Understaffing of veterinary personnel** | "You may find that one staff is caretaking two administrative units... in some instance; you actually find one staff caretaking two administrative units" (KII-NTO-23) | "A veterinary officer looks after like 4 sub-counties. For example, like now here, I can spend like 5 months without seeing the doctor" (FGD-LO-2, Kitagate) | **Both acknowledge severe human resource constraints** (18 references in codebook) |
| **Fear of quarantine as reporting disincentive** | "When you talk about Rift Valley fever, you spoil someone's market for the product... as a country, so that alone of course is a barrier" (KII-DTO-4) | "One of the things that may stop one from reporting is fear that the people in authority could put up a quarantine and stop the movement of animals" (FGD-LO, Rwetango) | **Economic sensitivity universally recognized** (5 references in codebook) |
| **Confusion with other diseases** | "All the RVF cases that we have confirmed have been submitted as suspects of Ebola not Rift Valley fever" (KII-ACAD-2) | "I didn't know rift Valley disease causes miscarriages... I thought it was brucellosis" (FGD-LO, Rwetango) | **Diagnostic confusion acknowledged at all levels** (14 references) |
| **Value of community structures** | "We have these village team members, these are the foot soldiers put in the villages who could be given information to pass on to the community" (KII-NTO-7) | "We have those chances. Us as LC ones, we meet every month and discuss and exchange knowledge. We meet there with the veterinary officers and we report" (FGD-LCL-4, Kikagate) | **Both recognize LC system and VHTs as critical assets** |
| **b) Areas of Divergence (Differences)** | | | |
| **Absence of local RVF name** | "The word Rift Valley Fever is in English... Community members should have a name... it is very challenging to make it... to explain it to the community" (KII-ACAD-1) | "Most people don't know; the disease doesn't have a local name, when you say RVF; it sounds new and I can be confused with fever or malaria" (FGD participant) | **KIIs frame as linguistic/cultural barrier to communication; FGDs experience it as daily confusion** |
| **Feedback mechanisms** | "It been not easy because when someone report, they should get action and feedback. So, in terms of motivation; most technical people have been demotivated" (KII-FAO) | "We report, but we never hear what happened" (Multiple FGD participants) | **KIIs discuss systemic demotivation of technical staff; FGDs express personal disillusionment** |
| **One Health coordination** | "The most challenging part where we have not strengthened is the 'one health approach' because these are zoonotic infections which are under veterinary. So, for us; we only come in when… on the human part" (KII-38M) | No equivalent mention in FGDs | **One Health is a KII-level concern; community members experience the fragmentation but don't name it** |
| **Laboratory capacity** | "We need to have RDTs at point of care diagnostic so that we are able to discern... we need to have significant investment in the mosquito vector surveillance side" (KII-NTO-2) | "Laboratories are far and this also contributes to our failure to vaccinate because we're not sure of the disease. The laboratory costs are expensive" (FGD-LO-9, Endiizi) | **KIIs discuss technical solutions and infrastructure; FGDs experience distance and cost barriers** |
| **Staff motivation** | "Some laboratory personnel... they say why should I go ahead to get this sample when I am not facilitated" (KII-NTO-14) | "The challenges we face in fighting the rift Valley fever disease is that the veterinary doctors are not well paid and this they are not motivated to work" (FGD-LO-4, Rwetango) | **KIIs describe internal demotivation; FGDs observe and are affected by it externally** |
| **Policy barriers** | "The challenge we have as a country with RVF is that it falls into 2 Ministries; we have MAAIF and MoH" (KII-42M) | "We do not have laws. We do not have by-laws that force people to report. If we had those laws to force people to report... we do not have" (FGD-LCL, Kikagate) | **KIIs identify inter-ministerial coordination failures; FGDs identify absence of local enforcement mechanisms** |
| **Economic impact framing** | "This is a disease of economic importance; this a disease that can affect international trade... one of our backbone is actually farming" (KII-NTO-17) | "Animals are used for many things, they are used for paying dowry and bride price so that means even people can't marry or get married because there is a quarantine" (FGD-LO) | **KIIs frame at macroeconomic/national trade level; FGDs frame at household/cultural livelihood level** |
| **Trust in government** | "When you talk about Rift Valley fever, you are spoiling someone's market... that alone is a barrier because when you are trying to do the ideal" (KII-34F) | "The doctors should come and prevent us from this disease. Vaccination is surely good... but the doctors are not there" (FGD, Ngarama) | **KIIs acknowledge political sensitivity; FGDs express resigned acceptance of government absence** |
| **Self-treatment practices** | "Farmers' self-diagnosis and management... bypasses formal reporting channels" (Codebook | "Many people try to treat animals by themselves and do not give doctors a chance to do their job... they try to treat diseases which sometimes they give inappropriate medication" (FGD-LO-6, Nakivale) | **KIIs see as surveillance gap; FGDs see as necessity due to inaccessible services** |
| **Vaccination barriers** | "It is more of an attitude and the government, the farmers, the stakeholders should sit on table and ask themselves that question; why are we not vaccinating against RVF?" (KII-UVRI) | "When a disease breaks out, we look for the medicine and we can't find, we call doctors, and they don't have. Even those that sell drugs sell them expensively... and not every farmer can manage" (FGD-LO-4, Rugaga) | **KIIs frame as policy/attitude problem; FGDs frame as access/affordability proble** |

**Unique Insights from Each Group**

**Unique KII Insights (Strategic/Systemic Level):**

- Inter-ministerial coordination failures (MAAIF vs MoH)
- One Health platform weaknesses
- National laboratory capacity and hub system
- Policy inertia and political sensitivity around disease declaration
- International trade implications
- Surveillance system architecture (indicator vs event-based)
- Vaccine development and research pipeline

**Unique FGD Insights (Experiential/Community Level):**

- Daily reality of inaccessible veterinarians
- Self-medication practices as a survival strategy
- Cultural dimensions (dowry, bride price) of livestock loss
- Physical terrain and network connectivity barriers
- Distrust born from repeated reports
- Observation of demotivated staff
- Confusion between RVF and endemic diseases in practice
- Economic desperation leading to high-risk behaviours
