# Supplementary material for: Facilitators and barriers to community-based livestock abortion reporting for Rift Valley Fever surveillance in Uganda: A COM-B analysis
Source: PLoS One. 2026 Oct 8;21(10):e0359872. doi: 10.1371/journal.pone.0359872 (PMC13649006; doi:10.1371/journal.pone.0359872)
Supplement: Supplementary file 6 [file pone.0359872.s006.docx]

**S5 Appendix. Ranking of Facilitators and Barriers: Ranking of Barriers (Highest to Lowest Priority)**

| **Rank** | **Barrier** | **COM-B Domain** | **Quotes/Files** | **References** | **Justification** |
| --- | --- | --- | --- | --- | --- |
| 1 | **Poor knowledge about RVF among communities** | Psychological Capability | 22 | 55 | **Most cited barrier (55 references)** . KIIs confirm: "all confirmed RVF cases submitted as Ebola suspects." FGDs: "We don't know the disease." Foundational barrier—without recognition, no reporting occurs |
| 2 | **Few staff, big district (understaffing)** | Physical Capability | 8 | 18 | Severe human resource constraint. One vet for 4+ sub-counties. FGDs: "5 months without seeing the doctor." KIIs confirm no replacements for retired staff. Directly affects response capacity. |
| 3 | **Absence of feedback mechanisms** | Reflective Motivation | 3 | 12 | **Most demotivating factor** despite moderate references. KIIs: "when someone reports, they should get action and feedback... most technical people have been demotivated." FGDs repeatedly: "we report, but we never hear what happened." Creates vicious cycle of disengagement. |
| 4 | **Limited transportation means** | Physical Opportunity | 8 | 13 | Surveillance staff lack vehicles; shared vehicles recalled mid-mission. KII: "this specific vehicle may be needed for other surveillance activities." Directly impedes outbreak investigation |
| 5 | **Confusion of RVF with other diseases** | Psychological Capability | 10 | 14 | "All RVF cases confirmed were submitted as Ebola suspects." FGDs confuse with brucellosis, malaria. Absence of local name compounds problem. |
| 6 | **Economic sensitivity/fear of quarantine** | Reflective Motivation | 4 | 5 | "When you talk about RVF, you spoil someone's market." Powerful disincentive that overrides civic duty. Farmers sell sick animals quietly rather than report. |
| 7 | **Limited laboratory capacity at district level** | Physical Capability | 6 | 7 | Samples must travel to UVRI/NADDEC. Delays of days to weeks. No local diagnostic confirmation capacity. |
| 8 | **Poor One Health coordination** | Social Opportunity | 4 | 7 | KIIs: "the most challenging part where we have not strengthened is the 'one health approach'." Human and animal health sectors operate in silos. |
| 9 | **No local name for RVF** | Psychological Capability | 1 | 1 | Single reference but profound implication. KII: "community cannot label it... unless the community understands, they cannot change." Prevents disease entering community discourse. |
| 10 | **Farmers' self-diagnosis and management** | Reflective Motivation | 5 | 7 | Bypasses formal channels. FGDs: "people were used to buying drugs from shops." Perceived as faster than waiting for unresponsive system. |
| 11 | **Rough terrain and geographic remoteness** | Physical Opportunity | 6 | 9 | FGDs: "when you call a doctor, he overcharges you because he is coming from very far." Roads impassable in rain; isolates communities. |
| 12 | **Weak policies and regulations** | Psychological Capability | 7 | 8 | FGDs: "we do not have by-laws that force people to report." Unregulated animal movements across porous borders. |
| 13 | **Delays in reporting** | Physical Opportunity | 3 | 6 | FGDs: "a farmer says let me first wait and see." Passive surveillance mindset; reactive rather than proactive. |
| 14 | **Limited storage capacity (cold chain)** | Physical Opportunity | 3 | 4 | Vaccines and samples require cold chain; fridges inadequate or shared with human health programs. |
| 15 | **Low perceived risk during inter-epidemic periods** | Reflective Motivation | 0 | 0 | Mentioned in discussion; complacency sets in when no active outbreak. |
| 16 | **Distrust in government response** | Reflective Motivation | 0 | 0 | Implicit throughout; explicit in discussion but not coded separately. |
|  | **Ranking of Facilitators (Highest to Lowest Priority)** | | | | |
| 1 | **Presence of trained staff (VHTs, CAHWs, district teams)** | Physical Capability | 13 | 19 | Most cited facilitator. KIIs: trained 70+ IPC focal persons, 30+ lab officers. FGDs acknowledge accessible vets as motivator. Foundation of surveillance capacity. |
| 2 | **Mobile phones and communication platforms (WhatsApp, radio)** | Physical Capability | 11 | 13 | FGDs: "We have phones and we can call the vet." KIIs: district health WhatsApp groups. Ubiquitous technology enables reporting without new infrastructure. |
| 3 | **Community structures (LC1 meetings, VHTs, local leaders)** | Social Opportunity | 5 | 9 | FGDs: "we meet every month and discuss... we meet there with the veterinarians and report." Existing governance mechanisms provide reporting pathways. |
| 4 | **Knowledge about RVF (from training/experience)** | Psychological Capability | 20 | 29 | Trained farmers recognize signs. KIIs: "farmers who had received training were better able to identify abortions as a potential sign of RVF." Modifiable capability. |
| 5 | **Presence of national reference laboratories (UVRI, CPHL, NADDEC)** | Physical Opportunity | 5 | 8 | Confirmatory diagnosis capability exists at national level. Turn-around time ~24 hours once samples arrive. Critical for outbreak confirmation |
| 6 | **Fear of economic and cultural losses** | Automatic Motivation | 5 | 8 | FGDs: "significant economic losses due to death and abortion." Livestock for dowry creates powerful intrinsic motivation to seek help. |
| 7 | **Emergency Operations Centre (EOC) capacity** | Physical Capability | 1 | 1 | KII: "when we receive an alert in the emergency operations centre, then we verify the information." Coordination hub for response. |
| 8 | **Experience from past outbreaks** | Reflective Motivation | 6 | 10 | Communities remember severity. KII: "preparedness Uganda... has done so well in generally outbreaks." Collective memory motivates reporting. |
| 9 | **Decentralized surveillance system** | Physical Opportunity | 3 | 3 | Focal persons at district, HSD, facility levels. KII: "right from the health facility; we have facility surveillance focal persons." Structure exists despite resource gaps. |
| 10 | **One Health coordination (where functioning)** | Social Opportunity | 7 | 8 | KII: "we supported joint risk assessment in 2019, and country prioritized RVF." Policy-level commitment exists; implementation at district level is weaker. |
| 11 | **Presence of cold chain system** | Physical Opportunity | 5 | 7 | KII: "we have fridges though they are not enough." Basic infrastructure for sample preservation and potential vaccination exists. |
| 12 | **M-Trac platform and electronic reporting** | Physical Opportunity | 1 | 2 | Mobile-based reporting system for human health; potential for integration. KII: "officers can now send in immediate mobile reports." |
| 13 | **RVF as priority zoonotic disease** | Social Opportunity | 3 | 3 | Policy recognition attracts attention and potential resources. KII: "the country prioritized RVF among the three diseases. |
| 14 | **NGO support (FAO, Red Cross, medical teams)** | Social Opportunity | 4 | 6 | Fill gaps where government resources insufficient. Training, transport, surveillance support. |
| 15 | **Diagnostic innovation capacity** | Physical Capability | 1 | 1 | KII: "we are developing an open platform where we are able to design a diagnostic using PCR." Local research capacity for future solutions. |
