# Supplementary material for: Facilitators and barriers to community-based livestock abortion reporting for Rift Valley Fever surveillance in Uganda: A COM-B analysis
Source: PLoS One. 2026 Oct 8;21(10):e0359872. doi: 10.1371/journal.pone.0359872 (PMC13649006; doi:10.1371/journal.pone.0359872)
Supplement: Supplementary file 7 [file pone.0359872.s003.docx]

**S2 Appendix. The FGD guide used during Focus Group Discussions**
